# Influence of openings on the snowdrift characteristics of cubes

**DOI:** 10.1371/journal.pone.0325545

**Published:** 2025-06-18

**Authors:** Xintong Jiang, Hanbo Cui, Shenghao Guo, Zongyun Mo

**Affiliations:** 1 School of Architecture and Civil Engineering, Anhui Polytechnic University, Wuhu, China; 2 Engineering Research Center of Anhui Green Building and Digital Construction, Anhui Polytechnic University, Wuhu, China; 3 School of Materials Science and Engineering, Chang’an University, Xi’an, China; ICIMOD: International Centre for Integrated Mountain Development, NEPAL

## Abstract

Snow drifts and accumulates under wind actions, leading to a complex distribution of snow around and on the surface of structures with openings, which in turn has a detrimental influence on the structures. There is no relevant code provision for this situation. Therefore, a wind tunnel study was carried out to experimentally investigate how opening size and vertical location (for cubes with single openings) and how the relative positions of different openings (for cubes with multiple openings) influenced the wind-induced snowdrifts around and on the surface of cubes with openings. A roughness coefficient was introduced to characterize the snow around cubes. It was found that snow accumulation at the side of the cube produced a greater uneven snow load. The number and vertical location of openings were the main factors influencing the roughness of the snow. The snow depth coefficient (*C*_*s*_) around and on the surface of a cube is approximately negatively correlated with opening size and approximately positively correlated with opening vertical location. The simultaneous existence of openings on the windward, side and top faces of a cube adversely affected the safety of the surroundings of the cube. The variation pattern of fractal characteristic of particles, *D*, was similar to that of *C*_*s*_ when the snow was fully eroded at the windward corner of the cube. These findings will help improve the accuracy of snow distribution pattern prediction and enhance the safety and rationality of the structural design of buildings.

## Introduction

Wind-induced snow drift is the result of the interaction between wind and snow particles [1–3], and the interaction between wind and saltation is often viewed as consisting of four subprocesses: aerodynamic entrainment, particle trajectory, splash entrainment, and air flow modification [4]. Thus, snow is prone to accumulate around the perimeter of buildings and on surfaces. Meanwhile, in wind induced snow drift studies, large opening structures usually refer to a class of structures with significant openings or unenclosed spaces, where the size the opening or the opening ratio (the proportion of the opening area to the overall surface area) is large enough to cause the wind field to form a complex flow pattern inside and outside structure, thus significantly affecting the distribution and drift behavior of snow. For instance, the J1 teaching building of Shandong University of Science and Technology, large openings were opened in the center of the structure (floors 1th-3th). The Water Church is a one-story building in Hokkaido, Japan. The front opening is 15m × 15m × 5m, nearly the area of the front wall. Such structures are more sensitive to the coupling effect of wind and snow due to interference at the openings, and snow is easily deposited on the sidewalls of the structures, which hinders the use of doors, windows, and evacuation routes. There is an urgent need to clarify the mechanism by which openings affect the pattern of snow around and on the surface of structures with openings to more accurately predict the snow distribution and develop and improve the relevant specifications.

The spatial and temporal variability of snowcover has been extensively studied because snow drift have a strong impact on avalanches and ecology. Overney [5] and Lieberherr [6] applied a Lagrangian stochastic model driven by the Large Eddy Simulation (LES) flow field to describe snow transport on small spatial scales and short time scales. Groot Zwaaftink et al. [7] found that a spatiotemporally varying snow mass flux can be determined if particle aerodynamic entrainment is driven by shear stresses acquired from the LES. The most reliable way to study wind-induced snowdrift is to conduct wind tunnel tests. The test results can assist with theoretical research, verify the accuracy of numerical simulations, and allow the adjust the test parameters better than field measurements. To simulate the natural snow drifting environment, it is necessary to make the parameters of the wind tunnel tests as much like the real-world situation as possible, which has been investigated by a number of experts. Strom et al. [8], Odar [9], and Anno [10] pointed out that wind tunnel tests on wind-induced snowdrift should conform to similarity laws in terms of geometry, kinematics, dynamics and should satisfy important parameters such as particle scale, coefficient of restitution, Froude number, and speed ratio. Anno [10] further noted that the angle of repose and the size of particles are also important similarity parameters. Liu et al. [11] conducted a scale test based on the similarity parameters proposed by Anno [10] and Lever and Haehnel [12] found that the application of the similarity criterion based on the mass transfer rate of the saltation process can accurately reproduce the snow pattern measured in the field.

Limited by the test conditions, materials similar to snow particles must be used in wind tunnel tests. The various parameters (e.g., mean particle size, angle of repose, density, threshold speed, settling speed, and Reynolds number) of fine quartz sand particles can simulate those of snow particles well [13–15]. The snow characteristics on the surfaces of different types of structures under multiple influencing factors have been investigated by a number of scholars, Liu et al. [16], Cao et al. [17] and Zhou et al. [18] have considered roof slope as a factor and reached some extremely important conclusions. Yu et al. [19] examined the influences of model size, wind speed, and wind blowing time on the snow drifting characteristics using a three-dimensional flat roof as a model. Wang et al. [20] studied stepped roofs through wind tunnel tests and numerical simulations and accurately predicted the distribution of snow on the roofs. Zhang et al. [21] compared the snow distribution on complex long-span structures under different wind inflow directions through wind tunnel tests and decomposed the snow on the roof into several basic snow distributions based on empirical orthogonal functions. Jiang et al. [22] took adjacent large-span stadium and gymnasium with openings as research objects and analyzed the influences of the stadium opening direction, spacing and wind direction angle on the characteristics of snow around the building. Using gable roofs with scuttles as a research object, Liu et al. [23] concluded that wind speed and roof pitches are important factors affecting the uneven snow distribution on the roof, and shields or other projections on the roof influence the snow distribution. To realistically simulate natural snow drifting, Zhang et al. [24] chose a new similarity criterion (including test wind speed, snowfall intensity, and transported snow profile) for wind tunnel tests, and the test results were in good agreement with the measured results. Jin et al. [25] conducted tests on two adjacent cubes in an open-air combined snow-wind experimental facility and analyzed the influences of wind direction angle, wind speed, and spacing on the snow distribution around the structures.

There is currently no suitable physical quantity to describe the pattern of snow in the surrounding area of a structure. Mandelbrot [26] introduced the concept of fractal theory to quantitatively characterize the geometry, composition, and structural surface profile of irregular objects. The application of fractal theory to the study of the surface roughness characteristics of porous media such as coal [27], granite [28], and concrete [29] has matured. Experts have carried out preliminary explorations in the field of turbulence using this theory. Balkissoon et al. [30] studied the fractal characteristics of wind speeds at three different towers in Missouri, USA and found that the time series determined by multifractal analysis was larger than that by single-fractal analysis. Yan et al. [31] calculated the fractal dimension as a quantitative indicator of the persistence of wind speed time series. Based on fractal theory, Shu et al. [32] investigated the variation in the fractal characteristics of vertical wind speed.

In summary, the direction of these studies is primarily centered around the snow distribution on the roof, rarely investigate the snow distribution pattern around the structure, and even more rarely do studies model structures with openings. However, the wind flow trajectories around open and closed structures differ significantly. The presence of openings leads to a partial suction of incoming wind, causing internal winds to flow leeward through these openings. These distinct wind flow patterns ultimately induce changes in the trajectory of snow particles, resulting in a complex snow distribution on and around the structure. Directly applying the snow distribution pattern of closed structures to the design of open structures clearly deviates from the actual project conditions. This deviation poses substantial safety risks. In addition, most of the previous descriptions of the test results use the snow depth coefficient of a single measuring point to reflect the snow distribution of a structure, which cannot capture the influence of the uneven distribution of snow on the surroundings of the structure. To address this problem, we took cubes with openings as research objects and systematically analyzed the influence of different opening factors (size, vertical location, number and relative positions of openings) on the snow distribution around and on the surface of the structure. We applied fractal theory to analyze the roughness characteristics of snow particles around the structure and used the roughness coefficient to describe the unevenness of the snow distribution around the structure. The particle fractal characteristics can be used as an important indicator of the degree of influence of different opening factors on the snow distribution, providing a new method for scientifically characterizing the snow distribution.

## Materials and methods

Similarity theory has been well applied in the field of wind-induced snow redistribution experimental research, therefore, this article will not go into detail here. The main aspects considered in the experiment were the geometric similarity of test model, angle of repose similarity of snow particles, kinematic similarity of the atmospheric boundary layer, the similarity of the aerodynamic drag, Reynolds number similarity and time-scale similarity [33–36].

The fractal dimension is defined in many ways and has a wide range of applications, and no unified standard has come to be. Common fractal dimensions include the Hausdorff dimension, box dimension, similarity dimension, and association dimension.

The length of a fractal curve is defined as:


$$\delta \left(x\right)=L_{0}x^{1-D}$$ (1)

where is a function of criterion . The smaller is, the more pronounced the measured roughness and the more tortuous the structure line. Therefore, in fractal dimension measurement, it is important to ensure that is small enough to obtain the true value of . There is a critical criterion or a critical number of generation steps such that the obtained fractal dimension approaches the true value.

At present, there is no fractal dimension formula for the roughness of snow particles around a structure. Through comparison, we found that the creeping motion of particles is similar to the surface morphology of rock joints, both of which are natural fractals and have a high degree of roughness. Therefore, the formula proposed by Xie and Pariseau [37] was used to calculate the fractal dimension of snow particles:


$$D=\frac{\mathrm{lg}4}{\mathrm{lg}(2(1+\cos(\arctan(\frac{2\bar{h}}{B}))))}$$ (2)

where *D* is the fractal dimension, *B* is the average base length, is the average height:


$$\bar{h}=\frac{1}{n}\sum_{i=1}^{n}h$$ (3)

where *n* is the number of measuring points on the longitudinal axis, h is the particle depth.

### Overview of wind tunnel tests

**Fig 1:**
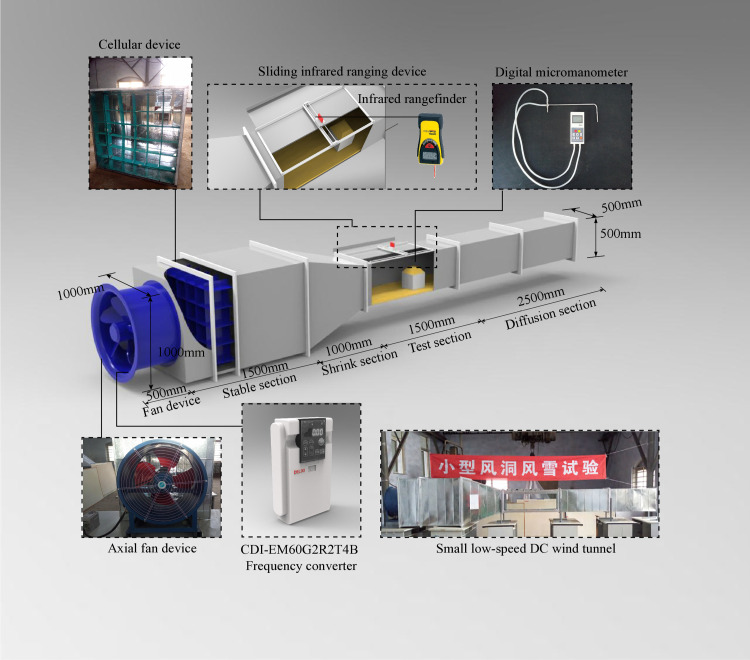
Small-scale low-speed open-circuit wind tunnel.


The wind tunnel tests were carried out in a Small-scale Low-speed Open-circuit Wind Tunnel [38]. The dimensions and equipment of each section of the wind tunnel are shown in Fig 1. The test section had dimensions of 0.5 m × 0.5 m. The relationship between the wind speed *V* and the frequency *f* of fan was fitted into formula (4). The fitting coefficient $R^{2}=0.979$, when the fan frequency was below 5 Hz, wind speed in the test section changed imperceptibly, the dynamic pressure fluctuation was small, and the uniformity and stability of the flow field were significantly reduced, the wind field quality of the test section was poor, so the stable wind speed of 1.5–10 m/s. The independent *Re* of the wind tunnel was determined through the parameter sensitivity analysis and stability verification as 6.30 × 10^4^. The test platform choosed to simulate the atmospheric boundary layer wind field by erecting the tower tips and controlling the frequency of the fan device. An airflow stability and the dynamic pressure field coefficient were satisfy the Building Engineering Wind Tunnel Standard requirements [39]. The flow field data for the original wind speed of 5 m/s, corresponding to a wind speed of 4.83 m/s, were measured at a roof height of 10 cm and using a sampling time of 3 mins. The measured results of the mean wind speed and turbulence intensity profile both meet the requirements [38].

V=0.199f+0.837(5Hz≤f≤50Hz) (4)

### Test content

**Fig 2:**
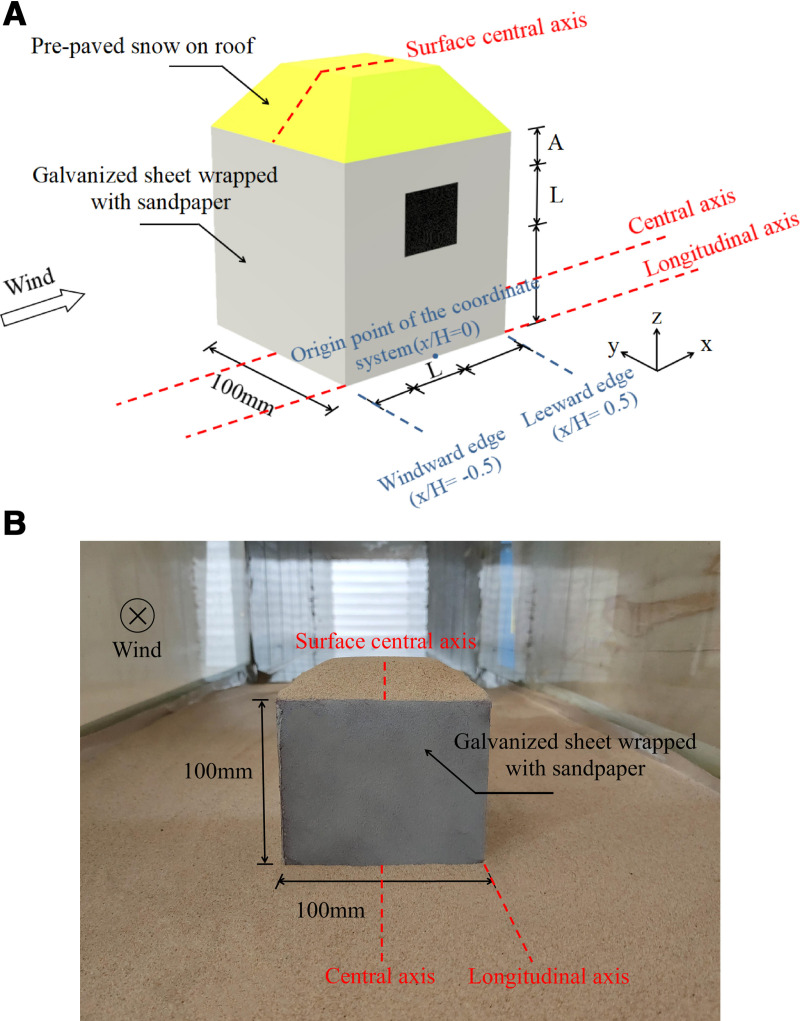
Model diagram of cube with opening. A) Axonometric drawing. B) Morphological graph.

The specific steps for test preparation were as follows:

(1) Selection of test model: The model was a cube structure, made of 2 mm thick galvanized sheet, to increase the contact area of particles, the galvanized sheet is wrapped with sandpaper. The cube had dimensions of 100 mm × 100 mm × 100 mm. Because of the relationship between the wind field characteristics, the distribution shape of snowfall, and the axis, the distribution of snow around the cube is analyzed taking the longitudinal axis, and the distribution of snow on the cube surface is analyzed by taking the surface center axis, as shown in Fig 2.

(2) Determination of the basic properties of the quartz sand particles layer: The particle size of the selected quartz sand was measured by sieving method, and the mean particle size was found to be 0.14 mm, the particle size of real snow particles in nature is between 0.15–1.0 mm [33,40,41]. The particles were measured to have an angle of repose of 30.8°, and a density of 2.56 $g/cm^{3}$. The similarity parameters, such as threshold speed $u_{*t}$, $u_{*t}^{3}/2gv$, and $u_{*}/u_{*t}$, all met the requirements. However, there was a large difference in the Froude number because the settling speed $w_{f}$ of the model was higher than that of the prototype. This is due to the Froude number is the ratio of the inertial force to the gravity, the settling speed $w_{f}$ of snow particles is usually determined by the equilibrium conditions of the particles in the fluid. In practice, high wind speed or small particle size result in Froude number increased, promoted snow drifting. But Anno [10] and Beyers et al. [40] analyzed the similarity parameters of the model and the prototype and concluded that the requirement for the Froude number can be appropriately reduced by considering the Reynolds number. And it has been confirmed that quartz sand particles can simulate the characteristics of snow particles (e.g., mean particle size, angle of repose, density, threshold speed, settling speed, and Reynolds number) [13–15].
(3) Method for laying quartz sand particles layer: The pre-paved particle method is a static simulation test, which required the determination of the initial snow depth and blowing rate [42]. The inflow area of the test model was covered with a layer of quartz sand particles of 0.3 m × 0.5 m, passive devices such as vertical tower tips and roughness elements were set up. This method can accelerate boundary layer development. A simple box of 0.5 m × 0.5 m × 1 m was placed at the lower end of the wind tunnel outlet to collect the quartz sand particles layer blown out of the wind tunnel so as to calculate their mass transport rate, the calculated value of the snow transport rate was between 0.328–0.585.
(4) Determination of test time, speed, and laid quartz sand particle thickness: The natural snow drifting process was simulated according to the field measured climate data by Oikawa et al. [43], using a ground roughness of class A, The wind speed could change at any time during the actual measurement. The test wind speed was determined to be 4.5 m/s considering the similarity criterion and the measured mean wind speed. The test time was set to 5 mins according to the time scale similarity.
(5) Selection of test parameters: The snow depth coefficient *C*_*s*_ is used to reflect the changes in the surface morphology of the snow cover. *C*_*s*_, as a dimensionless parameter, which is the dimensionless transformation of the final depth using the initial depth of particles as the characteristic scale.

$$C_{s}=h/h^{\prime }$$ (5)

Where *h* is the final depth of the quartz sand particles after the test is stopped, $h^{\prime }$ is the initial depth of the particles. Because the pre-paved particle method is adopted, $h^{\prime }$ is taken as 20 mm.

The *C*_*s*_ is greater than 1 when particles are deposited, and less than 1 when particles are eroded. For the measurement of *h*, using a sliding infrared rangefinder, and the measurement process was as follows:

A Before the test, the laser rangefinder was placed vertically on the slide, the top cover of the wind tunnel was opened, the laser rangefinder was controlled through the slide to measure the height $h_{0}$ from the surface of the quartz sand particles layer at each measuring point to the rangefinder, and the top cover of the wind tunnel was closed for the test.
B After the test, the same method was used again to measure the height $h_{1}$ from the surface of quartz sand particles layer at each measuring point to the rangefinder. Finally, the depth of quartz sand particles layer was

$$h=20-(h_{1}-h_{0})$$ (6)

### Test scheme

Taking cubes with openings as the research objects, ‘openings’ are defined as structural discontinuities in building envelopes that permit bidirectional environmental interactions, including wind-driven snow penetration and aerodynamic pressure equilibration. These openings are divided into the operable openings and passive openings based on architectural practice, operable openings refers to doors and windows on the structural surface with adjustable closing state, passive openings refers to permanent apertures. We study the form of large openings in passive vents. It has a large proportion of the opening to the total surface area of the structure, or the geometric of the opening (such as span, height) is close to the overall size of the structure. Wind tunnel tests were conducted to study the snow distribution of cubes that had different sizes, vertical location, numbers and relative positions of openings. The size of the opening is selected based on the actual structure, and the area rate of the opening is between 15% − 65%. Since the research on the opening factor is relatively rare, the typical square opening section is preferred for analysis. The specific test scheme was as follows:

A A cube with opening was selected to investigate. To study the influence of the opening size L, the opening was always located on the windward face of the cube, with the center of the opening at the center of the windward face of the cube. The working conditions 1–6 corresponded to different L values, as shown in Table 1. To study the influence of the opening vertical location A (distance between the top of the opening and the roof), the opening was located on the windward face of the cube for the test. The opening had dimensions of 40 mm × 40 mm, with the distance from the left (right) wall of the opening to the left (right) wall of the cube set to 30 mm. The vertical location A of the opening was the distance from the upper wall of the opening to the upper edge of the side face of the cube, with working conditions 7–13 corresponding to different A values.
B A cube with openings on two faces was selected to investigate, the two openings were put in two different walls of the same cube. The size of each opening was set to 40 mm × 40 mm, working conditions 14–19 corresponded to windward and leeward faces, windward and side faces, windward and top faces, side and side faces, leeward and top faces, and side and top faces, respectively.
C A cube with openings on three faces at the same time was selected to further investigate, the openings were located on three different walls of the same cube. Under working conditions 20 and 21, two openings were located on opposite side faces; under working conditions 22 and 23, two openings were located on the windward and leeward faces, respectively; and under working condition 24, three openings were located on the windward, side, and top faces, respectively.

**Table 1:** Test scheme.

Working condition	Opening size L/mm	Opening Number	Opening vertical location A/mm	Opening relative position(s)
1	0 × 0	1	0	windward face
2	40 × 40	1	30	windward face
3	50 × 50	1	25	windward face
4	60 × 60	1	20	windward face
5	70 × 70	1	15	windward face
6	80 × 80	1	10	windward face
7	40 × 40	1	0	windward face
8	40 × 40	1	10	windward face
9	40 × 40	1	20	windward face
10	40 × 40	1	30	windward face
11	40 × 40	1	40	windward face
12	40 × 40	1	50	windward face
13	40 × 40	1	60	windward face
14	40 × 40	2	30	windward and leeward faces
15	40 × 40	2	30	windward and side faces
16	40 × 40	2	30	windward and top faces
17	40 × 40	2	30	two side faces
18	40 × 40	2	30	leeward and top faces
19	40 × 40	2	30	side and top faces
20	40 × 40	3	30	Windward, side and side faces
21	40 × 40	3	30	Top, side and side faces
22	40 × 40	3	30	Windward, leeward and side faces
23	40 × 40	3	30	Windward, leeward and top faces
24	40 × 40	3	30	Windward, side and top faces

The specific test scheme is shown in Table 1.

### Verification of accuracy

To compared the findings of the wind tunnel test values with the actual field measurements made by Oikawa et al. [43] in Hokkaido, as well as the results from Li et al. [44] an numerical simulation of snow accumulation around the cube, were chosen as references. Select the measured working conditions SN09 and SN36 of Oikawa for comparative verification. The actual measuring time of the SN09 and SN36 were 24h, and snow depth of 200 mm. The maximum wind speed of the SN09 was 4.3 m/s, average wind speed was 1.7 m/s, the average wind direction was −15° (Rotation in a clockwise direction perpendicular to the windward was +). The maximum wind speed of the SN36 was 5.4 m/s, average wind speed was 3.1 m/s, the average wind direction was 1°. Li performed numerical simulations using the standard model and RSM model, respectively, set the wind speed at the height of the top surface of the cube to 5 m/s. The wind tunnel test model was a cube, selected the geometric scale ratio of 1/10 based on the actual field measurements, the model size for wind tunnel test was 100 mm × 100 mm × 100 mm, the quartz sand particle thickness of 20 mm.

**Fig 3:**
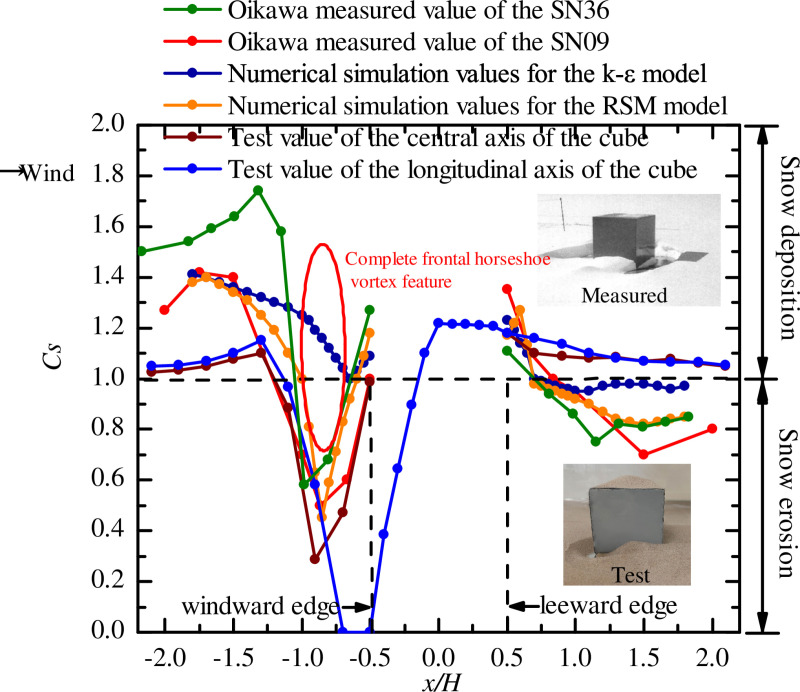
Comparison of snow distribution [45]. The dots represent the snow depth coefficient under field measurement, numerical simulation and wind tunnel test.

The horizontal coordinate *x/H* is the relative coordinate, which is the dimensionless transformation of the streamwise length *x* using the cube height *H* as the characteristic scale, *x/H* = −0.5 (*x/H* = 0.5) represents the windward (leeward) side of the cube, the vertical coordinate *C*_*s*_ is the snow depth coefficient. For the sake of comparison, *C*_*s*_ taken from the range of actual field measurements values *x/H* = −2.0 ~ 2.0, numerical simulation values *x/H* = −1.8 ~ 1.8, and wind tunnel test values *x/H* = −2.1 ~ 2.1. The wind-induced snow distribution on the longitudinal axis of the cube for the test and center axis for the other three operating conditions are shown in Fig 3.

Apart from the longitudinal axis cube working condition, comparison of other several working conditions in the windward region of the cube, it was found that the *C*_*s*_ tend to increase, then decreased and then increased. This variation in snowpack was due to the entrainment effect on the windward side, the most significant at *x/H* = −0.5 ~ −1.0. The longitudinal axis condition had different snow accumulation variation and *C*_*s*_ value with other conditions because of different measuring points selected on the axis. But it was the two snow distributions which reflected the generation of the horseshoe vortices at the windward edge of the cube. *C*_*s,*min_ values ranged from 0.45 to 1 (*x/H* = −0.87 ~ −0.65) for field measurements and numerical simulations, and 0.285 to 0 (*x/H* = −0.9 ~ −0.5) for wind tunnel test. The wind tunnel tests showed a decreased in the values compared to the field measurements and numerical simulations obtained by moving the vortex position downstream of the windward direction. In the leeward region, *C*_*s*_ varies in the form of ripples, the particles undergo creeping motion, but with varying values. It is believed that the reasons for the above differences are as follows: Firstly, the wind direction during field measurements varies randomly, the other working conditions are constant and vertical wind blowing. Secondly, inaccuracies caused by the instrumentation used in the measurements and tests, the material of the snow particles, and the human factors. Finally, the differences in the location of the measuring points lead to differences in the values of the *C*_*s*_. Consequently, it can be assumed that the Small-scale Low-speed Open-circuit Wind Tunnel can be used for the study of wind-induced snow distribution.

## Results

### Opening size

**Fig 4:**
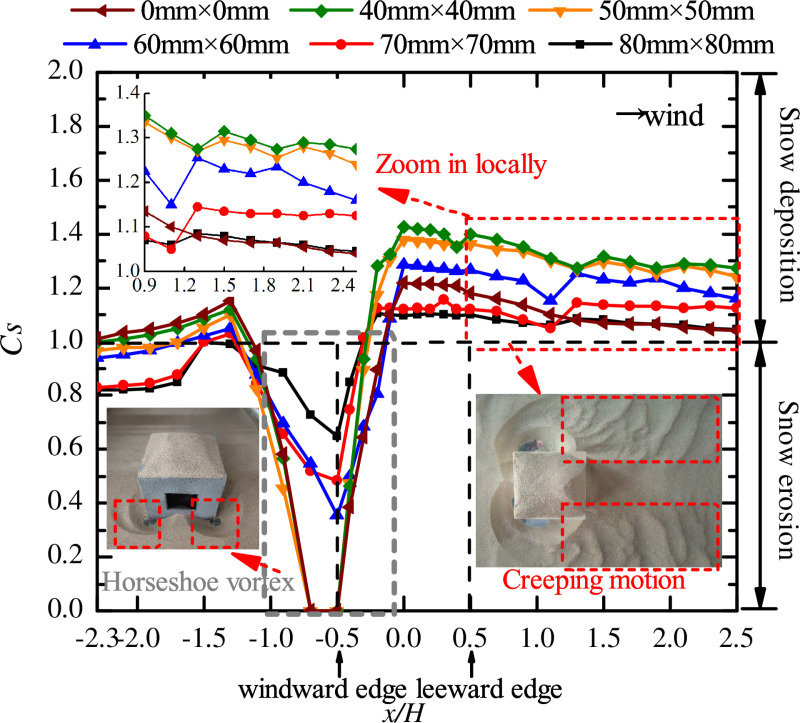
Curves of snow particle around cube with different opening sizes. The dots in the figure represent the snow depth coefficients on the longitudinal axis of the windward area, the edge of the cube side, and the longitudinal axis of the leeward area for different opening sizes (0 mm × 0 mm, 40 mm × 40 mm, 50 mm × 50 mm, 60 mm × 60 mm, 70 mm × 70 mm, 80 mm × 80 mm).

**Fig 5:**
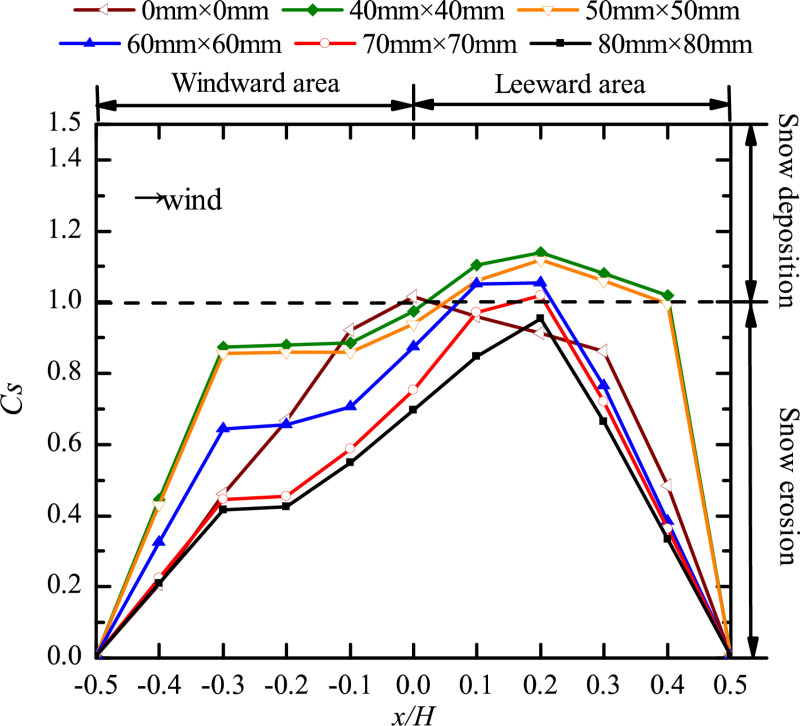
Curves of snow particle on the surface of the cube with different opening sizes. The dots in the figure represent the snow depth coefficients on the central axis of the cube surface for different opening sizes (0 mm × 0 mm, 40 mm × 40 mm, 50 mm × 50 mm, 60 mm × 60 mm, 70 mm × 70 mm, 80 mm × 80 mm).

Wind tunnel tests were carried out to obtain the curves of snow particle around (Fig 4) and on the surface (Fig 5) of cube.

Comparing the snow distribution curves of cubes with openings under five working conditions with no-opening condition (Fig 4), found that the snow depth coefficient *C*_*s*_ around the cube was greatly influenced by the opening factor. The particles in the leeward area were all deposited, with a large amount of deposition and *C*_*s*_ value between 1.1 and 1.4. Creep refers to the rolling and sliding of particles on the surface of the snowpack, where the height of the particles leaving the snow surface does not 10 mm during motion. The data in the red box area of the data in Fig. 4 (x/H=0.5\ 2.5), *Cs* first decreased, then increased, and decreased again, the motion form of the particle is in line with the characteristics of creep motion. In high wind speed or fine particle environment, saltation and suspension become dominant, and the role of creeping is significantly weakened. So, creep is not applicable to all wind speeds but is mainly present in the low to medium wind speed range, and its importance increases with coarser particles or higherhesion of the surface.

At the corners of the cube’s windward front (the gray box area in the Fig 4), a classic horseshoe vortex was generated. At the location of x/H=−0.5, the *C*_*s*_ was 0 with opening sizes of 40 mm × 40 mm, 50 mm × 50 mm, and no opening, respectively. As the opening size increased from 60 mm × 60 mm to 80 mm × 80 mm, the *C*_*s*_ at the lowest point of the vortex (x/H=−0.5) gradually increased; in the area of x/H=−0.9\ −0.5, the decreasing trend of *C*_*s*_ gradually slowed; and *C*_*s*_ increased slowly in the area of x/H=−0.5\ −0.1. Therefore, when the size of the opening was larger than 60 mm × 60 mm, the horseshoe vortex gradually shrank as the opening size increased.

In the windward area (x/H=−2.3\ −1.3), *C*_*s*_ showed an increasing trend, being greatest under the no-opening condition, and it was always greater than 1, that is, the particles were deposited. With the opening size of 80 mm × 80 mm, *C*_*s*_ was the smallest and was always less than 1, indicating that the particles were eroded. In the side area (x/H=−0.1\ 0.5) and the leeward area (x/H=0.5\ 2.5) of the cube, *C*_*s*_ decreased with increasing opening size. The location with the greatest difference in particle deposition depth was at x/H=0, the value of *C*_*s*_ was 1.425 under the condition of 40 mm × 40 mm at this time, and the value of *C*_*s*_ under the condition of 80 mm × 80 mm was 1.095. The *C*_*s*_ value for the 40 mm × 40 mm was 1.30 times that of the 80 mm × 80mms value. In summary, the opening size has a strong effect on the distribution of snow around the cube.

Fig 5 shows that there were three types of snow distribution on the surface of the cube with different opening sizes. The snow distribution patterns were the same with opening sizes of 40 mm × 40 mm and 50 mm × 50 mm, respectively, which were the most unfavourable to the surface stability of the cube. Snow was deposited in the leeward area, and *C*_*s*_ reached its maximum at x/H=0.2 with values of 1.14 and 1.12, respectively. The snow distribution patterns were the same with opening sizes of 60 mm × 60 mm, 70 mm × 70 mm, and 80 mm × 80 mm, and the value of *C*_*s*_ at the same measuring point decreased with larger opening size; when the opening size was 80 mm × 80 mm, the snow on the surface of the cube was all eroded, which was beneficial to the surface stability of the structure. When the cube had no opening, the snow distribution pattern on the surface of the structure was different from the above two patterns, and the most unfavorable measuring point of *C*_*s*_ was located at x/H=0, and its value was 1.015, indicating that the presence of an opening had a great influence on the distribution of snow on the surface of the cube. By comparing the snow distribution patterns of cubes, we found that the *C*_*s*_ of the same measuring point decreased with increasing opening size, as consistent with the snow particle curves variation around the cube.

### Opening vertical location

**Fig 6:**
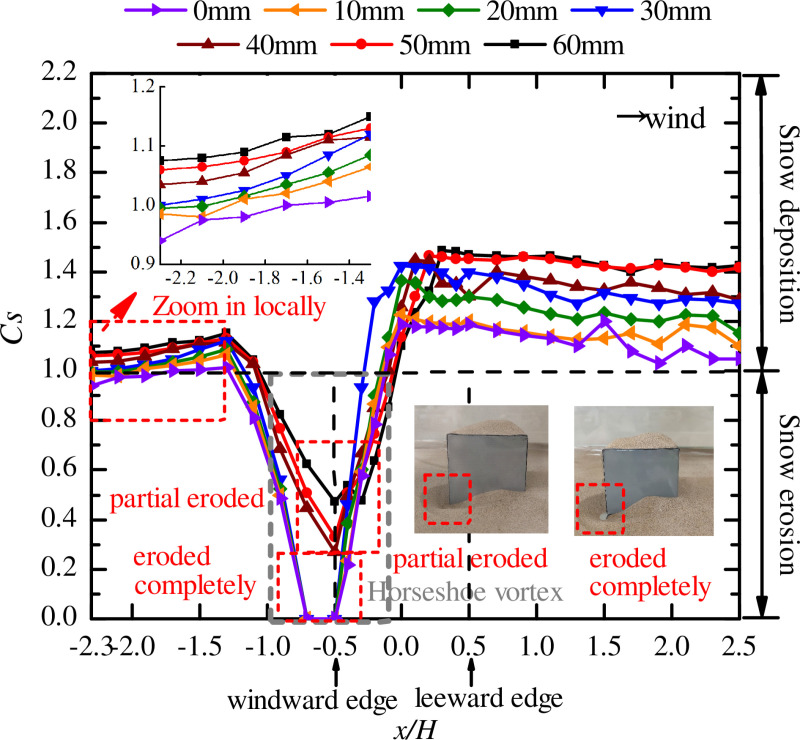
Curves of snow particle around the cube with different opening vertical locations. The dots in the figure represent the snow depth coefficients on the longitudinal axis of the windward area, the edge of the cube side, and the longitudinal axis of the leeward area for different vertical locations (0mm-60mm, each condition increased by 10mm).

**Fig 7:**
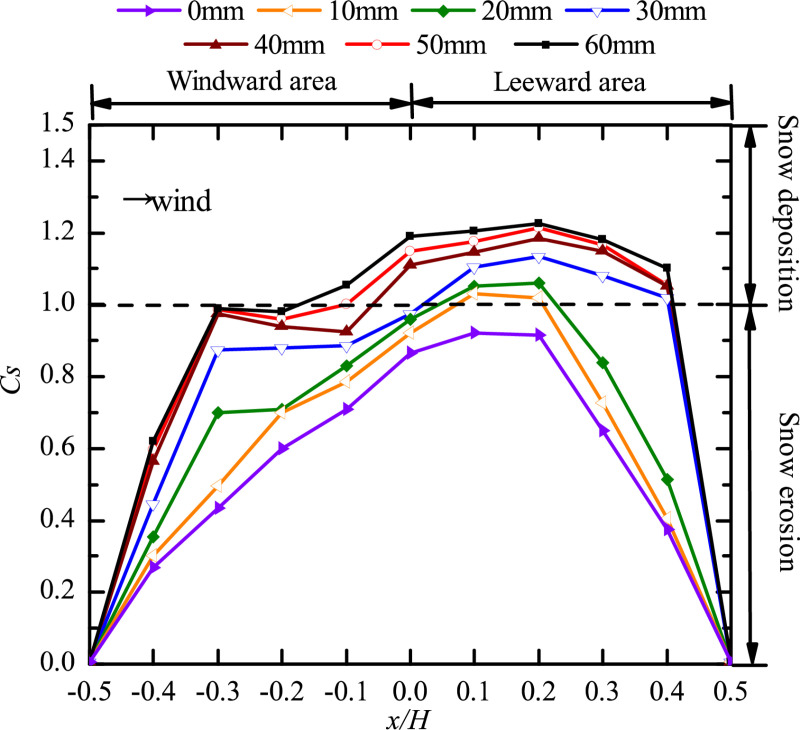
Curves of snow particle on the surface of the cube with different opening vertical locations. The dots in the figure represent the snow depth coefficients on the central axis of the cube surface for different opening sizes (0mm-60mm, each condition increased by 10mm).

As the vertical location of the opening on the windward face of the cube changed, so did the wind speed and direction. Fig 6 shows the curves of snow particle around the cube with different opening vertical locations obtained from the wind tunnel tests, and Fig 7 shows the curves of snow particle on the surface of the cube with different opening vertical locations.

In Fig 6, in the range of x/H=−2.3\ −1.3, *C*_*s*_ were increased for all seven working conditions, and *C*_*s*_ of the same measuring point increased with the amplify in the opening vertical location. The reason is that when the opening was a low, the pressure difference near the opening was large, which caused the wind speed to increase and resulted in some particles moving downstream of the windward side with the wind speed. By observing the horseshoe vortex (the gray box area in the Fig 6), we found that when the opening vertical location was 0 mm, 10 mm, 20 mm, or 30 mm, the particles were eroded completely and *C*_*s*_ was 0. When the opening vertical location was 40 mm, 50 mm, or 60 mm, respectively, only some of the particles were eroded, *C*_*s*_ at the center of the horseshoe vortex (x/H=−0.5) increased with the decrease in the opening vertical location, and the area of the horseshoe vortex was smaller than that under the four working conditions with opening vertical locations of 0 mm, 10 mm, 20 mm, and 30 mm, respectively.

A comparison with the peak values of *C*_*s*_ on the side face of the cube showed that under the four working conditions with opening vertical locations of 0 mm, 10 mm, 20 mm, and 30 mm, the peak value of *C*_*s*_ was located at x/H=0. When the opening vertical location was 40 mm, 50 mm, or 60 mm, the peak value of *C*_*s*_ moved backward at x/H=0.1, 0.2 and 0.3, respectively. The peak value of *C*_*s*_ decreased as the opening vertical location decreased, and the peak value of *C*_*s*_ at an opening vertical location of 60 mm was approximately 1.25 times that at 0 mm. In the leeward edge and leeward area of the cube, the maximum value of *Cs* around the structure was at opening vertical location of the 60 mm, followed by 50 mm, then 40 mm, decreased in turn, and the opening vertical location of 0 mm reached the lowest, *C*_*s*_ decreased as the opening vertical location decreased. In future engineering designs, openings in the low part of a building should be minimized.

Fig 7 shows that when the opening vertical location was 0 mm or 10 mm, the snow distribution on the surface of the cube had the same pattern, and the snow was eroded over a large area. Therefore, the above two designs help improve the snow distribution of the structure. When the opening vertical location was between 30 mm and 60 mm, the snow distribution on the surface of the cube was consistent. When the opening vertical locations were 50 mm and 60 mm, show was deposited in the area of x/H=−0.1\ 0.4, the maximum values of *C*_*s*_ were 1.215 and 1.225, respectively, and the resulting uneven snow load adversely affected the surface of the cube. When the opening vertical location was 20 mm, the snow distribution on the surface of the cube was between the above two patterns, there were two peaks of *C*_*s*_, and this distribution pattern was close to that corresponding to an opening vertical location between 30 mm and 60 mm. However, in the area of x/H=0.2\ 0.5, the variation trend of *C*_*s*_ was the same as that corresponding to opening vertical locations of 0 mm and 10 mm, and the value of *C*_*s*_ gradually decreased downwind. By comparing the snow distribution patterns under different opening vertical locations, we conclude that the *C*_*s*_ on the surface of the cube at the same measuring point decreased with the decrease in the opening vertical location, similar to the variation pattern of the snow around the cube.

### Position of two openings

**Fig 8:**
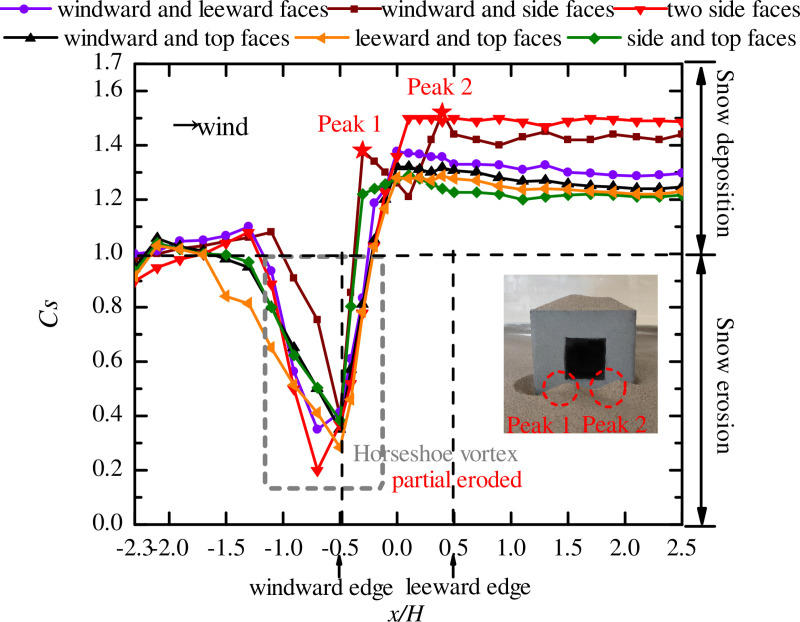
Curves of snow particle around the cube with openings on two faces. The dots in the figure represent the snow depth coefficients on the longitudinal axis of the windward area, the edge of the cube side, and the longitudinal axis of the leeward area for different positions of two openings (windward and leeward faces, windward and side faces, two side faces, windward and top faces, leeward and top faces, side and top faces).

**Fig 9:**
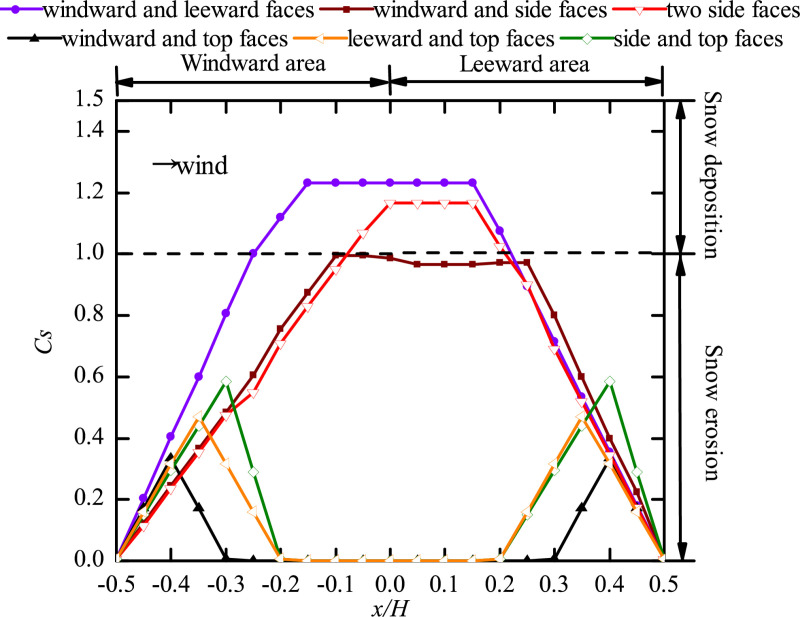
Curves of snow particle on the surface of the cube with openings on two faces. The dots in the figure represent the snow depth coefficients on the central axis of the cube surface for different positions of two openings (windward and leeward faces, windward and side faces, two side faces, windward and top faces, leeward and top faces, side and top faces).

To clarify the snow distribution when the openings on two different faces, the pattern of snow distribution around the cube (Fig 8) and on the cube surface (Fig 9) with different opening positions is discussed.

Comparison of Figs 4, 6, and 8 shows that the value of *C*_*s*_ at the center of the horseshoe vortex (x/H=−0.5) ranged from 0 to 0.65 when the cube had only one opening (Figs 4 and 6) and ranged from 0.2 to 0.4 when the cube had two openings (Fig 8). The $C_{s,\;\max}$ for the side face of the cube was between 1.1 and 1.465 when the cube had only one opening and $C_{s,\;\max}$ was between 1.26 and 1.5 when the cube had two openings. We conclude that the pattern of snow distribution around the cube varies significantly as the number of openings in the cube increase.

As shown in Fig 8, when the openings were located on the windward and side faces, the particle distribution on the side face of the cube was different from that under other working conditions. This was because the wind at the opening on the side face had a complex motion, where the incoming wind entered directly through the opening on the windward face and part of it exited through the opening on the side face, and the airflow around the cube also entered the structure through this opening, resulting in two peaks of *C*_*s*_ at the side face: peak 1 was located at x/H=−0.3 and had a value of 1.38, and peak 2 was located at x/H=0.4 and had a value of 1.52. The particle distribution in this case was extremely nonuniform, and the *C*_*s*_ was large, which adversely affected the safety of the side face of the cube. When openings were on two side faces at the same time, there was no opening on the windward face, and the incoming wind was blocked by the windward face to form two backflow vortices directly. The horseshoe vortex produced here had a large area, the particle erosion was the greatest, and the wind formed convection at the opening on the side face and increased the flow velocity, resulting in the greatest particle depth being in the side face and leeward area of the cube. At x/H=1.1 the *C*_*s*_ of the openings on the two side faces was 19.19% higher than that of the side and top faces. When openings were located in the side and top faces, the *C*_*s*_ of the side face and the leeward area of the structure was the smallest, and the particles were distributed uniformly, which was less dangerous to the structure. These findings suggest that structural designs should avoid having openings on the windward and side faces at the same time or on two side faces at the same time.

Fig 9 shows that when the cube had an opening on the top face, the snow distribution pattern was different from that under other working conditions, only a small amount of snow existed in the windward area (x/H=−0.5\ −0.2) and the leeward area (x/H=0.2\ 0.5), the snow was distributed in a triangular shape, and the snow distribution pattern was the same in the two areas. Comparing the three working conditions considered for an opening on the top face, we found that the snow on the surface of the cube was eroded under each condition, and *C*_*s*_ was the largest when openings were located on the side and top faces but had little influence on the stability of the cube. A comparison of working conditions revealed that when the openings were located on both windward and leeward faces, the snow was deposited in the area of x/H=−0.25\ 0.2, and the *C*_*s*_ value was kept to 1.23 in the area of x/H=−0.15\ 0.15, which was the most detrimental to the surface stability of the structure. When openings were made on side faces at the same time, snow deposition also occurred in the area of x/H=−0.05\ 0.2, and was 1.165 in the area of x/H=0\ 0.15, which was approximately 15.45% higher than that when openings were located on the windward and side faces. Therefore, when two openings are made at the same time in a building, it is important to avoid having the openings on opposite wall faces (windward and leeward faces, or opposing side faces). This conclusion is different from what we concluded about the variation pattern of snow around the cube. Therefore, when two openings are made in the cube, the snow distribution around and on the surface of the cube should be analyzed separately.

### Position of three openings

**Fig 10:**
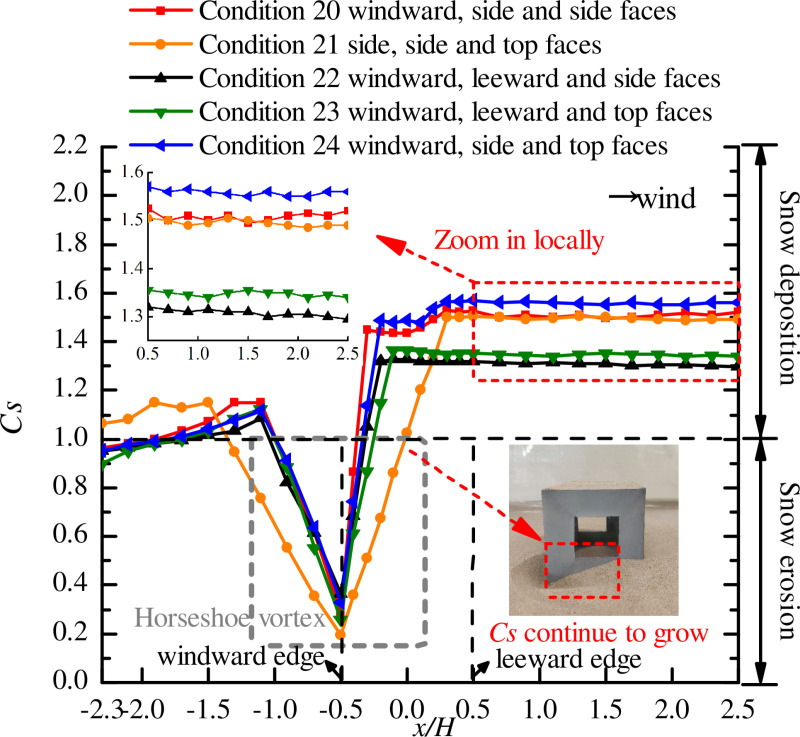
Curves of snow particle around the cube with openings on three faces. The dots in the figure represent the snow depth coefficients on the longitudinal axis of the windward area, the edge of the cube side, and the longitudinal axis of the leeward area for different positions of three openings (windward, side and side faces; side, side and top faces; windward, leeward and side faces; windward, leeward and top faces; windward, side and top faces).

**Fig 11:**
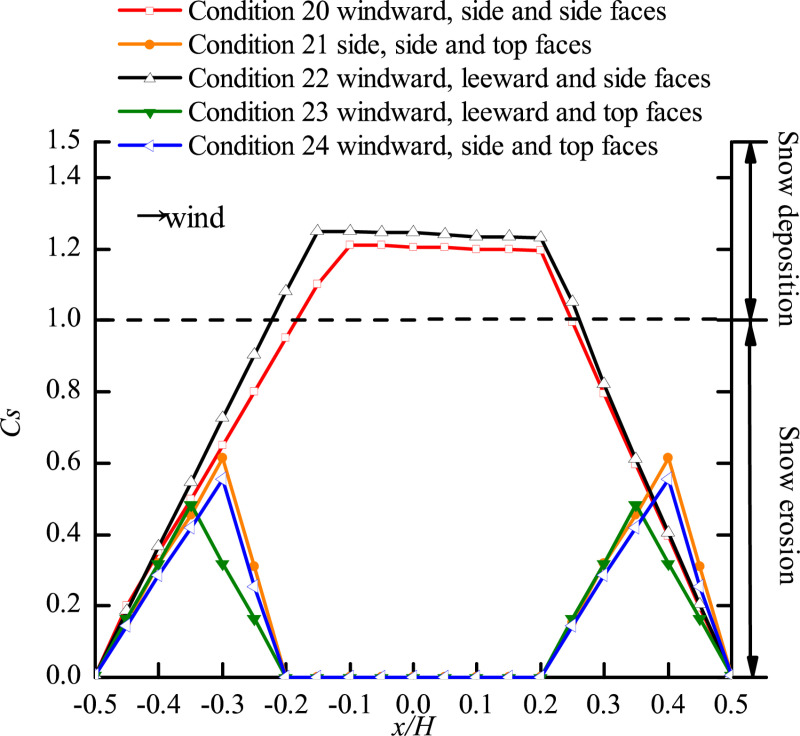
Curves of snow particle on the surface of the cube with openings on three faces. The dots in the figure represent the snow depth coefficients on the central axis for different positions of three openings (windward, side and side faces; side, side and top faces; windward, leeward and side faces; windward, leeward and top faces; windward, side and top faces).

When the cube had three openings, the opening positions are described in Section 3.3. The curves of snow particle around and on the surface of the cube with openings on three faces are shown in Figs 10 and 11, respectively.

A comparison of Fig 8 (two openings) with Fig 10 (three openings) showed that the distribution of particles around the cube varied with the number of openings, especially when the locations and values of $C_{s,\;\max}$ were different in the side face area of the cube (x/H=−0.5\ 0.5), thus verifying the conclusion in Section 4.3 that the number of openings in the cube had a significant impact on the snow distribution.

As shown in Fig 10, the side face of the cube, the snow distribution patterns under working conditions 24 and 20 were different from those under other working conditions, and there were two peaks of *C*_*s*_ on the wall of the cube, with the value of peak 2 larger than the value of peak 1. This phenomenon was the same as when the openings were located on the windward and side faces in Fig 8, and the cube had openings on both windward and side faces under all the three working conditions. The snow distribution around the cube under working condition 21 was completely different from that under other working conditions. In the windward area of the cube (x/H=−2.3\ −1.5), *C*_*s*_ increased with fluctuation, and the area of the horseshoe vortex at the windward corner reached its peak value at the maximum value of *C*_*s*_, and the location of $C_{s,\;\max}$ on the side face (x/H=0.3) shifted backward significantly. In the area of x/H=−0.5\ 0.3, *C*_*s*_ increased linearly and then fluctuated between 1.485 and 1.5. Analysis of working conditions 20, 21, and 24 revealed that the snow distribution around the cube was the most unfavorable to the structural stability when the openings were located on the windward, side, and top faces. It can be seen that the presence of openings on the two side faces of the structure caused air convection and changed the snow distribution around the cube, thus adversely affecting the structure.

Analysis of the curves of snow under working conditions 22 and 23 showed that there was relatively uniform distribution of snow on the side face of the cube, where the value of *C*_*s*_ was smaller than that under the other three working conditions. The reason for this was that two of the openings were located on the windward and leeward faces of the cube, allowing the airflow to form convection in the direction of the incoming flow. At the same time, the convective vortex within the cube was altered at this location under the influence of the third opening, resulting in less snow on the side face of the cube.

Fig 11 shows that when one of the three openings of the cube was located on the top face (working conditions 21, 23, and 24), the snow on the surface of the cube was eroded and distributed in a triangular shape, and the snow distribution patterns in the two areas were the same. This was consistent with the snow distribution pattern on the surface of the cube with two openings (Fig 9). Analysis of the snow distribution curves under working conditions 20 and 22 shows that that snow was deposited in the area of x/H=−0.15\ 0.2 under both working conditions, with $C_{s,\;\max}$ being 1.21 and 1.245, respectively, which was unfavorable to the safety of the structure. Therefore, when there are three openings in a building at the same time, two openings should not be located on opposite wall faces (windward and leeward faces or opposing side faces).

### Fractal dimension

**Fig 12:**
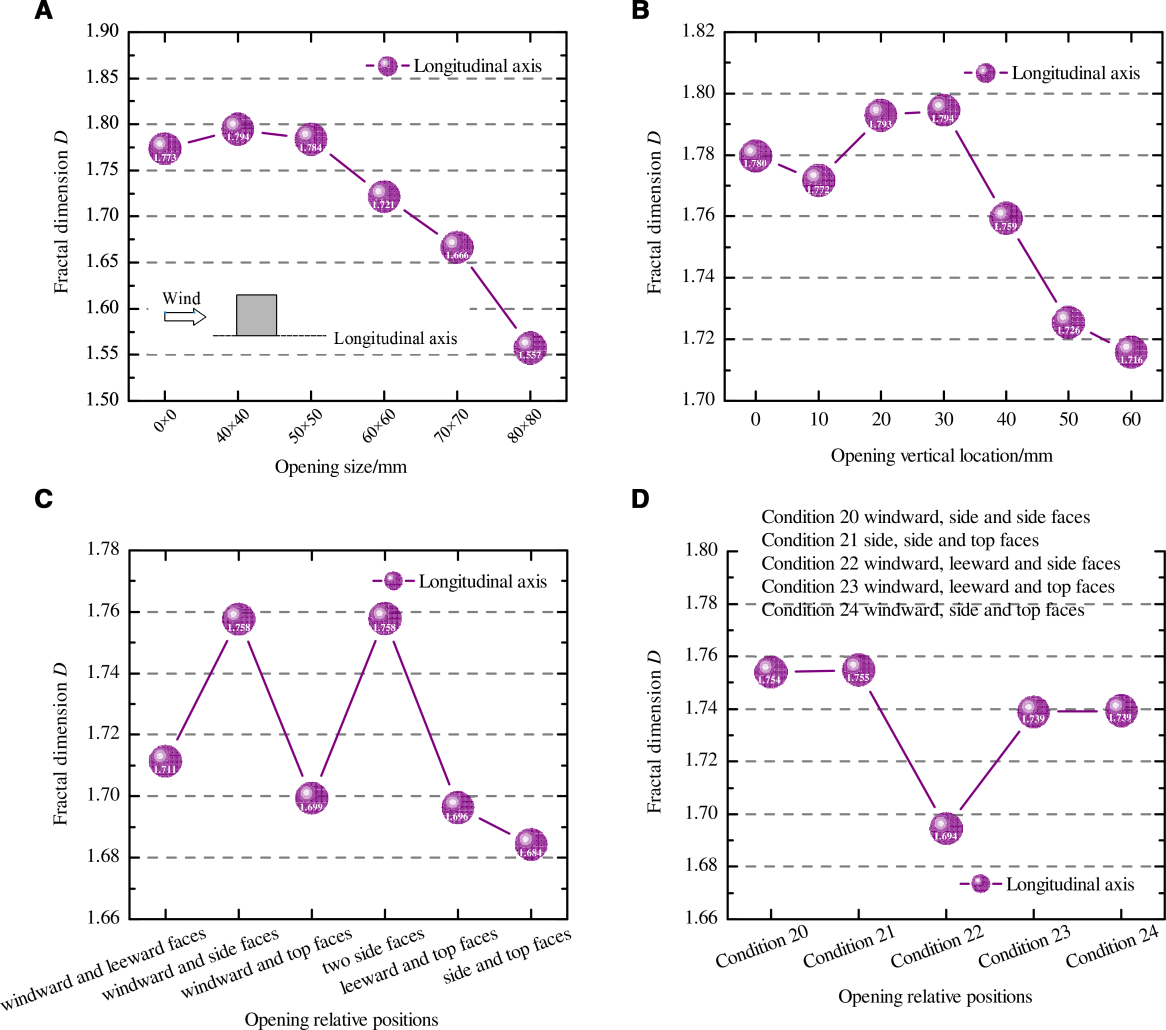
Variation patterns of particle fractal characteristics. (A) Opening size, (B) Opening vertical location, (C) The relative position of two openings, (D) The relative position of three openings. The dots in the figure represent the fractal dimension under different influencing factors. The figure (a) shows the fractal dimension when the opening size is 0 mm × 0 mm, 40 mm × 40 mm, 50 mm × 50 mm, 60 mm × 60 mm, 70 mm × 70 mm and 80 mm × 80 mm. The figure (b) shows the fractal dimension when the vertical location is 0-60 mm. The figure (c) shows the fractal dimension when the relative position of two openings is windward and leeward faces, windward and side faces, two side faces, windward and top faces, leeward and top faces, side and top faces. The figure (d) shows the fractal dimension when the relative position of three openings is windward, side and side faces; side, side and top faces; windward, leeward and side faces; windward, leeward and top faces; windward, side and top faces.

To study the fractal characteristics of particles along the longitudinal axis of the cube, we explored the variation in the fractal dimension (*D*) of particles as opening size, opening vertical location, the relative position of two openings, and the relative position of three openings changed (Fig 12). This parameter reflects the comprehensive morphological characteristics of the particle coverage area and can also determine the degree of influence of the four factors on the snow distribution pattern.

Comparing Fig 12A and Fig 4, it can be seen that under the five working conditions for openings, the change trend of the fractal characteristics of the particles along the longitudinal axis of the cube was consistent with the change in the snow depth coefficient of the surrounding particles, both showing a gradual decrease in *D* and *C*_*s*_ as the opening grew. *D* was 1.794 for the opening size of 40 mm × 40 mm, which is approximately 13.21% higher than that for the opening size of 80 mm × 80 mm.

As shown in Fig 12B, the *D* value (1.772 to 1.794) for opening vertical locations between 0 mm and 30 mm was significantly larger than the *D* value (1.716 to 1.759) for opening vertical locations between 40 mm and 60 mm, indicating that the particle distribution around the cube was less uniform under the first four working conditions. This pattern was opposite to that of the snow distribution around the cube shown in Fig 6. This was because when the opening vertical location of the cube was in the range of 0 mm to 30 mm, the particles were completely eroded, with a particle depth of 0 mm at the center of the horseshoe vortex on the windward front of the cube. When the opening vertical location was in the range of 40 mm to 60 mm, the depth of the particles was between 5.4 and 9.4 mm, resulting in the average height of the particles under the four working conditions with opening vertical location 0–30 mm being smaller than that under the other three working conditions. Therefore, the *D* value for opening vertical location between 0 and 30 mm was greater than that for opening vertical location between 40 and 60 mm.

Fig 8 shows that at the horseshoe vortex at the windward front of the cube, the particles were not completely eroded, and the *C*_*s*_ value was in the range of 0.28 to 0.415. Comparison with Fig 12C shows that under the conditions of different positions of two openings, the variation pattern of *D* along the longitudinal axis of the cube was similar to the distribution pattern of particles around the cube; Under the working conditions where openings were located in two opposing side faces of the cube as well as in the windward and side faces of the cube, *D* was 1.758, and the snow distribution around the structure was extremely nonuniform; and when openings were located on the side and top faces, *D* was 1.684, and this arrangement of openings was less hazardous to the structure.

According to Fig 12D, when openings were located on the side faces of the cube at the same time (conditions 20 and 21), *D* was the largest, which was about 1.04 times that under working condition 22. As shown in Fig 12A–12D, when the cube had only one opening, revealed that the *D* value for different opening vertical locations (1.716 to 1.794) was greater than that for different opening sizes (1.557 to 1.794). Therefore, the opening vertical location had a greater influence on the snow distribution of the cube than the opening size. When the number of openings was different, the *D* value varied between 1.694 and 1.755 in the case of three openings, with more complex snow distribution pattern and particle roughness characteristics.

## Discussion

The distribution pattern of wind-induced snowdrift of the cube was investigated to determine the variation in snow depth coefficient (*C*_*s*_) around the structure and on its surface under different factors. A fractal dimension (*D*) approach was introduced to explore the uneven distribution of snow particles in the surrounding area. Cs and *D* reflect different characteristics of snow, so the similarities and differences between the *C*_*s*_ and *D* of particles around the cube were explored in depth.

**Fig 13:**
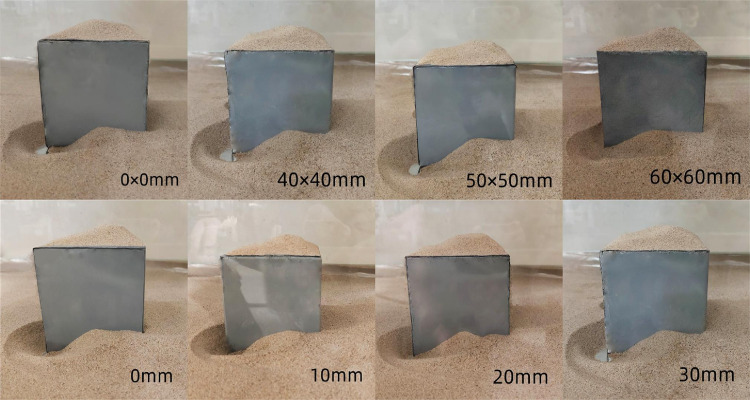
Particle distribution patterns. Effect of different opening sizes and opening vertical locations on the erosion degree of the horseshoe vortex located at the front of the cube.

The snow formed a horseshoe vortex at the windward corner of the cube, where the particles were somewhat or completely eroded, and there was a large height difference between the particles. Since *D* was calculated based on the average height *h* of the particles, whether the particles at the horseshoe vortex were completely eroded had a great influence on *D*. That is, the *D* value obtained when *C*_*s*_ at the horseshoe vortex was 0 was higher than that when *C*_*s*_ was not 0 (Fig 13). In the figure, the particles at the windward corner of the cube were completely eroded under three working conditions with opening sizes of 0 mm × 0 mm, 40 mm × 40 mm, and 50 mm × 50 mm, respectively, and the particles were partially eroded under other working conditions. Therefore, the *D* values under the three working conditions in Fig 12A were greater than those under other working conditions. Similarly, the *D* values under the three working conditions with the opening vertical location in the range of 0 mm to 30 mm in Fig 12B were larger than those under the other three heights. When the *C*_*s*_ values at the horseshoe vortex were the same, the overall roughness of the snow changed in the same pattern as the snow depth factor. When the snow at the windward corner of the cube was completely eroded, the fractal characteristics of the particles reflected the changes in the snow depth coefficient.

*C*_*s*_ mainly reflects the variation in the snow depth around the cube and has the advantages that the variation in snow depth can be observed in different areas (windward area, side face of the structure, and leeward area) and the specific values of snow depth at each measuring point can be obtained directly, allowing the location of the maximum snow depth to be obtained quickly. *D* focusses on the overall uneven distribution of the snow in the area near the structure, reflecting the fluctuating pattern of the snow. The fractal dimension describes the complexity and self-similarity of the surface geometry. The fractal dimension determines the scale dependence of the roughness, and the roughness parameter reflects the amplitude characteristics of the fractal surface. The combination of the two can the complete morphology of the surface from the micro to the macro. Its advantage is that the roughness coefficient can be used to indicate the unevenness of the overall snow distribution, but first it is necessary to determine the amount of erosion or deposition of the particles. The factors that are most detrimental to the overall distribution of snow on a structure can be determined by comparing the *D* values under different influencing factors. The larger the value of *D*, the rougher the particle coverage area around the structure, while *C*_*s*_ only reflects the absolute value of the snowdrift height. For this reason, in future research on wind-induced snowdrift distribution, *C*_*s*_ and *D* will be analyzed simultaneously to characterize the snow distribution around the structure more comprehensively. In the subsequent work, we will employ 3D visualization tools to generate intuitive representations of snowpack spatial distribution patterns, including 3D surface maps and volumetric renderings. Furthermore, will utilize 3D spatial analysis techniques to quantitatively characterize snow distribution features, such as snow surface roughness and accumulation density. This integrated approach will provide both visual and metric insights into the three-dimensional structural properties of snowpacks. Moreover, we will consider the characteristics of snow accumulation around and on the surface of the structure under the influence of coupling factors with wind direction angle, opening size, opening vertical location, number openings, and relative position of the openings as variables, the quantitative relationship between the different influencing factors of openings and the distribution of snow around the structure will be established, the mechanism by which the openings influence the snow particle motion trajectory will be elucidated, and the structural instability failure under the influence of opening factors (size, vertical location, number, relative positions of openings and wind direction angle) will be provided as a basis for research. Meanwhile, this paper mainly focuses on the study of snow accumulation characteristics (distribution patterns and roughness characteristics), and does not investigate the mechanical properties of the structure itself (e.g., strength, stability and dynamic response).

## Conclusions

Taking cubes with openings as the research object, wind tunnel tests were conducted to investigate the distribution patterns of snow on a cube under different numbers, size, vertical location, and arrangements of the openings, and to analyze the influences of several factors on the surface roughness characteristics of the particles. The specific conclusions are as follows:

(1) The *C*_*s*_ around (in the windward and leeward areas) and on the surface of the cube is approximately negatively correlated with opening size and approximately positively correlated with opening vertical location. Hence, it is advisable for the structural design to avoid incorporating excessively small openings in lower locations on the structure’s wall.
(2) When openings were located on the side and top faces of the cube at the same time, the snow distribution was favorable to the stability of the surroundings and surface of the structure; when openings were located on the windward, top, and side faces of the cube, the snow distribution was the most unfavorable for the safety of the surroundings of the structure. Arrangements of openings on both side faces at the same time should be avoided, under this condition, the structure will generate uneven snow pressure.
(3) The roughness of snow accumulation along the structure’s perimeter decreased as the opening size increased. The highest *D* values and the least favorable snow distribution along the perimeter occurred when the structure had openings on the windward side and one side or both sides simultaneously. As such, the simultaneous analysis of *C*_*s*_ and *D* in the study on a structure characterized the snow distribution more comprehensively and provided a reasonable basis for the design of structures with openings subjected to wind and snow.

## Supporting information

S1 File Experimental data. (XLS)
